# Foreign Summary

**Published:** 1839-06-15

**Authors:** 


### FOREIGN SUMMARY.

*On the Varieties and Treatment of Fractures of the Ribs.* By J. F. Malgaigne.—[The name of Malgaigne, attached to any essay, the subject of which is fracture or disturbance of bone, is sufficient to call attention to it. The author of the communication which affords the materials for the following analysis, has studied the subject in a comprehensive and scientific manner; and we doubt not that there is in the present paper not a little that will be novel and interesting to many of our readers.]

From a review of our knowledge on the subject of fractures of the ribs, M. Malgaigne concludes that the clinical and experimental history of this affection is still a desideratum; all which is at present taught in the schools being unsupported by anything like proof. The author says that his attempt will be to supply the necessary information; for which purpose he has studied the normal figure of the ribs, he has instituted experiments upon the corpse, has collected cases from the living, has procured pathological specimens, and has gathered from books such information as was available for his object.

*Causes of fractures of the ribs. External causes.* The opinion general maintained, that fracture of a rib takes place *almost always towards the middle of the rib*, is stated to be incorrect. M. Malgaigne says that the majority of such fractures are seated in the anterior half of the rib. Direct causes may produce their effects on all parts; but the anterior parts are the most exposed to their action, the posterior portions being protected by muscles and by the scapula; the middle, by the arm and the shoulder. And with respect to indirect causes, M. Malgaigne has very often tried to break the ribs by a sudden and forcible pressure on the sternum, but the fracture has always been in the anterior half, and generally nearer to the sternum than to the middle of the ribs. Several reasons may be given why this should be the case. The posterior extremities of these bones being more elevated than the anterior, if, for example, the heel is pressed upon the sternum, on a level with the insertion of the sixth rib, the pressure corresponds posteriorly almost with the level of the tenth rib. The first effect of the pressure upon the anterior extremity of the rib is to force it backwards and downwards simultaneously; that is to say, to diminish in one direction, but to increase in another, the interval which separates the extremities of the bone. When fracture takes place, therefore, it is not in consequence of simple increase of the curve, but because of the twisting which results from the depression of the anterior extremity. As this movement takes place especially in this extremity, it is quite natural that it should more particularly suffer. Again, the anterior pressure acts upon the sternum beyond the anterior extremity of the rib, prolonging the arch in this direction; but the posterior pressure acts particularly on that part of the bone which is just anterior to the angle, and which projects so much behind, that the body rests upon it in decubitus. Now, these two circumstances explain why the centre of the arch, the curve of which is increased by the fracturing force, is much anterior to the centre of the bone. And, lastly, anatomy indicates and experience reveals another reason of the fact above stated. Pressure does not act on all the ribs simultaneously; and those which are not pressed upon, supporting the others, prevent them from yielding as much as if they were isolated. Thus, for example, press with the hand upon the sternum, on a level with the sixth rib; the sternum sinks, and, at the same time, approaches the vertebræ. But, increase the pressure, the bone does not sink any further, and its superior extremity, held firmly by the ribs, remains almost immoveable, whilst the inferior is pressed towards the vertebræ. The ribs follow this movement unequally; the sixth rib, being more directly subjected to pressure, bends more; the seventh and the fifth, somewhat less, and so on. So that the point at which flexion commences varies with each rib, and, consequently, cannot be always the centre of the arch which they describe; and, lastly, this point of flexion cannot be very far separated from the sternum, because of the resistance of the neighbouring ribs. From this binding together of the ribs when they resist pressure on the sternum, it happens that in almost every case several ribs are simultaneously fractured, when the cause of such fracture is indirect; and, on the other hand, as these fractures always take place in the anterior half of these bones, a series of fractured ribs in the vicinity of the sternum, excepting where they may have been caused by the wheel of a carriage passing over the ribs themselves, are almost inevitably dependant on an indirect cause. Many individuals suffered fractures of the rib in an enormous crowd, assembled on the Champ de Mars, in 1837. Of twenty-three who died, seven had fractured ribs. The number of ribs which were broken, varied from two to thirteen in the same individual; and all the fractures were anterior, and between one inch and a half and two inches and a half from their cartilages. But a single rib may be broken by an indirect cause; in which case the pressure has acted solely upon the cartilage, or upon the extremity of this rib.

With regard to the *internal causes* of fracture of the ribs, we can here only allude to several cases, which M. Malgaigne has collected, of fracture taking place during cough, in cases where there does not appear to have been any peculiar fragility of bone. The individuals to whom the accident happened were all, however, somewhat advanced in years. Drs. Gooch and Graves are alluded to by the author as having published cases of this description. In a diagnostic point of view, the fact, possibly of less rare occurrence than is supposed, should not be lost sight of.

There are three principal kinds of fractured ribs: 1. *Incomplete fractures.* 2. *Simple fractures.* 3. *Multiple fractures.*

1. *Incomplete fractures.* This may occupy the inferior or superior half of the bone, or the internal or external surface. Fractures of the latter kind are simple or multiplied, most generally affecting the internal table, but sometimes the external alone. Direct or indirect causes produce them, and several ribs are commonly affected at the same time. These fractures are so readily produced, either upon the entire corpse, or upon a rib isolated and separated from the soft parts, that it is difficult to resist the inference that incomplete fractures of the ribs are of much more frequent occurrence than we appear justified, from our actual knowledge, in supposing them to be. Two causes may account for our inability to decide this doubt: the negligent mode of diagnosticating fractured rib, and the infrequency of autopsies. But there are cases of incomplete fracture on record, occupying the various situations already mentioned. Such cases are detailed by M. Malgaigne.

2. *Complete simple fractures.* These are either oblique or transverse, the fracture being clean: or they are very irregular, each fractured surface being covered with projecting points and angles.

3. *Multiple fractures.* These fractures, although scarcely recognised, are probably as frequent as the second variety. The double fracture is sometimes incomplete. Complete fracture may be associated with an incomplete fracture, or the fracture may be complete in two situations, or there may be three or even four fractures in the same rib. In the "Musée Dupuytren," two anatomical specimens are preserved, where several ribs are broken together; in one case, all the fractures are simple; in the other, they are double. Of nine anatomical specimens, in the possession of M. Malgaigne, five exhibit a consolidated simple fracture; two present double complete fractures of the same rib, the middle fragment being from three to four inches in length; one shows the traces of three fractures, the hindermost of which, close to the angle of the rib, appears to have been complete, and the other two, half an inch and four inches anteriorly, are incomplete. In the last specimen are traces of four fractures: one towards the angle of the rib, complete; a second incomplete, and half an inch more anterior; and others, more anterior still, which appear to have been complete. The callus of complete fractures may be readily distinguished, however small may have been the displacement: it surrounds the rib like a rough and projecting ring; whilst in incomplete fractures the external face (unbroken in all the specimens seen by M. Malgaigne) shows no vestige of bony deposit, and the imperfect ring of callus is only seen on the inner surface or on the borders of the bone.

*Displacements to which fractured ribs are subject.* In the *incomplete fractures*, when there is but a fissure in the bone, whether longitudinal or transverse, there is no displacement. M. Malgaigne broke off the inferior border of the rib with the blow of a hammer, and here there was displacement; and he has a specimen of a fracture of the internal table and diploe, effected by himself, the external table being simply somewhat depressed opposite the fracture, a depression which would probably escape observation on the living subject. But the most important circumstance in this specimen is, that the anterior fragment of the inner table projects inwards about a line, and that this projection cannot, by any movement, be replaced. By compressing the extremities of the ribs, so as to increase its curve, the internal fragment was in some degree replaced; but whilst increasing the pressure, so as to complete the reduction, the external table was broken, and the fracture then rendered complete. A similar result was attained from fracturing the external table and the diploe, without injuring the internal table. A fragment projected externally, which could not be reduced by any means. M. Malgaigne has an anatomical specimen representing, he thinks, this fracture; and he supposes that such an external projection might take place as to be evident, on examination, through the soft parts. The author forced in the seventh rib by a violent blow with a hammer. In the situation of the blow, an angular concavity could be felt, instead of a fracture: the internal table was broken in two points, separated from one another about two inches and a half, and the fragment resulting from this fracture was only adherent by its centre to the rib. Cheselden speaks of having found, in autopsies, upon the external surface of the ribs, an impression of the thumb and four fingers of nurses. It is supposed that the condition of parts may have resembled that just described. M. Malgaigne does not maintain that, even in multiple fractures of this kind, displacement always takes place. When the depression affects several ribs, as happens from the wheel of a carriage, the diagnosis is immediately evident. A depression of various extent and size exists; and if, in examining it with the fingers, no projection of any fragment is felt, if the pressure increases, for an instant, the depression, without producing any projection, the existence of an incomplete multiple fracture of the internal table may be diagnosticated.

In the *simple complete fractures*, there may often be no displacement, when, for instance, the periosteum is untorn, or the fracture very serrated; but displacement as often occurs, although, frequently, not to such a degree as to be perceptible through the soft parts. Of such displacement, M. Malgaigne has described examples in his possession. In one case the posterior fragment projects inwards for nearly a line, and upwards in about the same extent. In a second, the displacement is of the anterior fragment, downwards and backwards about a line. A third shows a projection of the posterior fragment outwards. In one specimen, preserved by Dupuytren, several ribs are affected with simple fracture; the fracture is oblique, from one border to the other, but in opposite directions, and the displacement varies in consequence; thus, in the first of the broken ribs, the anterior fragment projects upwards; in the second, it is depressed beneath the posterior; and in the third, the displacement is similar to the first. In a skeleton, some of the ribs of which had been fractured ddring life, at about four fingers' breadth from their cartilages, the appearances were as follows: The anterior fragment of the fifth was carried inwards and downwards, the superior interosseous space being evidently diminished backwards; the anterior fragments of the third and fourth were depressed inwards; there was no displacement of the second, and the fracture could only be estimated by the roughness of the callus. Others have noticed such union as clearly indicated displacement: some attributed this to the treatment employed, the pressure recommended by Petit. But this explanation is inadmissible, as evidence of displacement exists when no such treatment was employed. Similar displacements are effected by blows upon the sternum and ribs of the corpse—experiments which have been frequently made by M. Malgaigne.

*Multiple fractures.* These, when complete, sometimes occur without displacement; more commonly there is displacement of one of the fragments, the other remaining almost in place; and sometimes all the fragments are simultaneously displaced. M. Malgaigne regards external violence and the configuration of the fracture, as the causes of the displacement. An external shock, for instance, partly fractures a rib: it acts first by thrusting it inwards; a greater force breaks the internal table and diploe, the denticulated form of the fractured surfaces prevents the return of the rib to its original position, and hence there remains a depression of the unbroken external table of the bone. Is the fracture complete? If the fracture is transverse and smooth, there is commonly no displacement, the bone returning, by its elasticity, to its original situation. But exception must be made for fractures occurring very near the sternum; partly in consequence of the ligamentous attachment of the ribs to this bone, the anterior fragment moving inwards and outwards, and which, when it has been carried inwards, has not, in consequence of the articulation, the elasticity of the posterior fragment. The case is similar, where a broken portion has become bent by a second fracture, either complete or incomplete; there remains no elasticity by which it may regain its position. When the fracture is oblique, the direction of its obliquity commonly determines that of the displacement. The denticulated extremities of fractured ribs are the most frequent among the causes of continued displacement; but with regard to fractures near the sternum, a special cause of displacement in a certain direction exists, and which also tends to reproduce displacement when it has been remedied. Pressure upon the sternum depresses the sternal portion rather than the other, and this pressure tends also to carry it downwards, motion in the two directions sometimes coexisting. This (the sternal) portion being depressed, the posterior fragment projects simply because it remains in its place. Decubitus on the back, a circumstance well deserving the attention of the surgeon, augments this projection, the posterior fragment of the ribs being pushed forwards; and if the patient lie upon the fractured side, there is still greater projection. The nearer the fracture is to the sternum, the more evident are these circumstances, and most particularly in fracture of the cartilages. M. Malgaigne has found, in the last case, that by varying the pressure upon the ribs, the anterior or posterior fragment might be made to project; a fact from which he has derived a method of treatment, to be noticed.

The *diagnosis* must be inferred from what has been said concerning the kinds of displacement. It is frequently very difficult, and always requires very great care. There are some special causes of error, which should be borne in mind. The insertions of the obliquus descendens and serratus magnus muscles might give rise to the notion of displacement, in consequence of their abrupt projection beneath the finger, especially when pain causes any spasmodic contractions in these muscles; and in some subjects there are remarkable projections at the union of the cartilage with the bone of the rib.

*Treatment.* The treatment of fractured ribs is shown, by what has preceeded, to be less simple than most surgeons have conceived it to be. The fractures without displacement require only to be kept at rest; those with displacement, and which are not disposed to be displaced when reduced, require reduction, in addition; and when there is a tendency to displacement after reduction, there is a third indication to fulfil, *i. e.* to prevent such secondary displacement.

1. *Means of keeping the ribs immoveable.* The rules laid down for using the bandage for the trunk are, that it is indispensable when it alleviates pain caused by respiratory efforts; that when there are no such pains, it is needless to employ the bandage; and that if pain continues, notwithstanding its use, it is both useless and injurious. In individuals with a large chest and vigorous constitution, the circular bandage is safe. M. Malgaigne prefers the following mode of applying it. Surround the chest, first of all, with a common bandage, and apply over this a piece of cere-cloth, (sparadrap,) about three fingers broad, and sufficiently long to pass twice round the body. But in feeble individuals, with narrow chests, agitated by chronic coughs or paroxysms of asthma, the indication is to confine the constriction to the injured side; an indication which it is not easy to fulfil. Decubitus upon the injured side would be very useful, could it be borne: if not, a demi-cuirass, made by soaking a bandage in an amylaceous decoction, might fulfil the proposed indication. But on this point M. Malgaigne only throws out suggestions, not having made it the subject of experiment. But he tried, in the following manner, to limit the action of the thorax by bands of cere-cloth (sparadrap.) The commencement of one band was applied on a level with the anterior extremity of the seventh rib of the right side, thence passed around the left side of the thorax, beneath the left scapula, and over the right shoulder: from this point it was passed a second time around the left side of the thorax, ending on a level with the crista of the right ilium. The costal respiration of the left side was thus evidently impeded, whilst it continued quite free on the right side. It would appear that the left ribs might be much more directly acted upon, by surrounding them with an oblique bandage, the two ends of which should cross one another at the right hip; but in this case the anterior part of the bandage, by compressing the abdomen, would interfere materially with the diaphragmatic respiration, which it is very important in these cases properly to manage. Or again, one side of the thorax might be acted upon by means of the spring of a hernial truss, the sternum and the spine being points on which the spring should press. A strap passing over the opposite shoulder might be used to support this, and, if necessary, a large vertical splint might be placed between the centre of the spring and the convexity of the ribs. This apparatus is applicable for the fulfilment of another indication, hereafter to be noticed.

2. *Means of reducing the displaced fragments.* In simple or double fractures, with depression of one fragment, the indication may consist only in elevating the depressed portion. But in some cases there is an actual projection of the other fragment outwards, produced by the bad position of the patient; but change of position suffices to rectify this. With regard to the former indication, M. Malgaigne observes that he had frequently tried the experiment on the corpse, of pressing gently downwards the fragment which remained in its proper situation, until it came in contact with the depressed fragment. He found that the inequalities of the two broken surfaces fitted into each other; and that, on removing the pressure, the elasticity of the rib brought back into its right position the former fragment, bringing the depressed portion with it. To effect this, certain conditions are necessary: if the fracture occupies the middle of a true rib, or is further backward, it is of little consequence which is the depressed portion; if it is more anterior, the posterior fragment alone possesses sufficient elasticity to produce the above effect, so that, were this fragment itself depressed, it would not be readily elevated. With regard to the false ribs, whatever situation the fracture may occupy, the anterior fragment can only be elevated by means of the posterior. Fortunately, by virtue of this elasticity, the depression of the former is much more frequent than that of the latter. Two cases are related in support of these views of treatment, derived from experiment upon the corpse. In one of these, although the reduction was not accomplished, the manipulations caused a sudden and remarkable relief of pain, leading to the belief that some irritating portion of bone might have been removed from contact with the lung. Remarking on the cases alluded to, M. Malgaigne observes that it required but in a trifling degree to diminish depression of one fragment to cause an instantaneous cessation of most acute pain, very probably by disengaging the lung from a fragment of bone which was pricking and irritating it, and bringing back the projecting piece beneath the costal pleura. It is to these depressed portions of bone that may be attributed the acute pains and the visceral inflammations which sometimes accompany fractured ribs; and if it is remembered that, frequently, whether the fracture be complete or incomplete, the displacement may not appear at all externally, whilst there is a considerable prominence of a portion of the inner table of the bone, we may be disposed to regard this circumstance as of more importance than has hitherto been the case. Morbid anatomy confirms (although not with much proof) the above explanation. M. Malgaigne contends that the necessity is almost as great for removing fragments of bone from the lung, as for removing them when driven into the brain. He alludes to the various methods which have been suggested for effecting this object; and he suggests the following: to take a needle, covered like a tenaculum, to plunge it as far as the superior border of the depressed fragment, and thence to pass it over the inner surface, almost as far as the channel in which runs the intercostal artery, employing the instrument then as a simple elevator. The incision may be thus avoided; and such a puncture is very harmless.

3. *Means of preventing return of displacement.* In fractures near the sternum, there is actual danger of this occurrence; and its causes are decubitus upon the back, and particularly on the injured side. The twofold indication is to keep the healthy side of the thorax forwards, so that the fragment which is attached to the sternum may be drawn in the same direction, and to keep up a constant pressure upon the portion which projects, equal in amount to the resistance afforded by the elasticity of the rib. The former indication is quite fulfilled in serious cases, by decubitus on the healthy side; and then, also, the little disposition of the ribs to move would render the second almost useless. But in less important cases, where the patient wishes to move about, and to walk, the two indications are fulfilled simultaneously by a truss for hernia, with a long spring, one extremity of which presses posteriorly upon the projection of the ribs, on the sound side; the other anteriorly, upon the posterior fragment itself. To obviate the injurious effects of prolonged pressure, compresses may be employed.—*Brit. and For. Med. Rev., from Archives Générales de Médecine.*

---

*Cissampeline, a new vegetable base.* By A. Wiggers.—M. Wiggers announces that he has discovered in the root of *Cissampelos pareira*, a vegetable basis, which is obtained in the following manner. The root is boiled in successive quantities of water, acidulated with sulphuric acid, and carbonate of soda is added to the brown decoction. A precipitate is thus thrown down of a grayish-brown colour, which is washed and redissolved in water acidulated with sulphuric acid. The solution is boiled with animal charcoal, and after filtration, carbonate of soda is added. A dirty yellow precipitate falls, which is dried, pulverized, and then boiled with ether. A nearly colourless solution is obtained, and by distilling off the ether, the cissampeline is procured. To purify the substance completely, it is dissolved in weak acetic acid, and again thrown down by carbonate of soda whilst the solution is still warm; the precipitate is then washed and dried with care.

It is known that M. Feneulle, who is occupied with the analysis of the *Pareira brava*, has discovered the presence of a yellow bitter principle in which the active properties of the root seems to reside. Can this principle be the impure cissampeline?—(*Journal de Pharmacie*, January 1839,) *from Edin. Med. and Surg. Journ.*

---

*On the preparation of Gentianine.* By Professor Dulk de Konisberg.—The experiments of Tromsdorff and Leconte have demonstrated in a decisive manner that the gentianine prepared according to the directions of M. Henri, cannot be regarded as the active principle of gentian. I have found that the active bitter principle of this root may be isolated by the following process. Treat the coarse powder of the root with alcohol; distil off the alcohol, and dissolve the residue in water; filter the solution. The undissolved matter treated with ether furnishes a clear tincture, from which we obtain by evaporation the gentianine of M. Henri, quite insipid.

The aqueous solution has a very bitter taste, and must be fermented to separate the sugar, which cannot be easily done in any other manner. The liquid is then precipitated by the neutral acetate of lead; the precipitate is separated and thrown away. Into the bitter filtered liquor is poured the solution of the subacetate of lead, and a little ammonia, to precipitate the combination of vegetable matter with the oxide of lead; but care must be taken not to add too much ammonia, because this, as the stronger base, is apt to separate the vegetable matter from the oxide of lead. We thus obtain a yellow precipitate, which is washed with small quantities of water, for a large quantity decomposes the compound. The precipitate is then suspended in water, and decomposed by a stream of sulphuretted hydrogen gas. Filter and evaporate at a low temperature to dryness; treat the residue with alcohol of specific gravity 0.820, filter, and by evaporation is obtained a mass which contains no traces of crystallization.

This gentianine has a yellowish-brown colour; dried and triturated, it furnishes a yellow powder; it possesses the bitter taste of the root in a high degree. It is hygrometric, is almost insoluble in absolute alcohol, more soluble in common spirit, and very soluble in water. It reddens litmus; heated it melts, swells up, and burns without residue; it contains no azote. By its reaction and relation to bases it approaches the acids.—*Ibid.*

---

*Medicinal properties of the gray oxide of zinc.* By Professor Sementini.—From a series of experiments made on the white and gray oxides of zinc, (the latter discovered by himself,) Professor Sementini, of Naples, draws the following conclusions: 1. the oxide of zinc possesses tonic properties, which it derives from its soothing the irritability of the nervous system; it is also anti-spasmodic and sedative. 2. This has long been known, but the use of the medicine has been abandoned, from the inconstancy of its effects. 3. That inconstancy arises from the facility with which it absorbs carbonic acid, and hence passes to the state of a subsalt. 4. The *gray* oxide does not absorb the acid, and is therefore always of uniform strength. 5. As the properties of a tonic and a sedative coexist in it, it may be used with the greatest confidence in cases of *irritative debility.* The dose to begin with, is from a fourth to half a grain, which may be increased to four or six grains, by an addition of a quarter of a grain every second day.—*Giornale dell. Sc. Med. Chir.*, No. 45, *Marzo*, 1838., *from Brit. and For. Rev. Med.*

